# Supplementary material for: A Novel Carboxylesterase Derived from a Compost Metagenome Exhibiting High Stability and Activity towards High Salinity
Source: Genes (Basel). 2021 Jan 19;12(1):122. doi: 10.3390/genes12010122 (PMC7835964; doi:10.3390/genes12010122)
Supplement: Supplementary file 1 [file genes-12-00122-s001.pdf]

Supplementary Figures and Tables

"A novel carboxylesterase derived from a compost metagenome exhibiting high stability and activity towards high salinity"

Mingji Lu[1], Rolf Daniel[1*]

[1] Department of Genomic and Applied Microbiology and Göttingen Genomics Laboratory, Institute of Microbiology and Genetics, Georg-August-University of Göttingen, Göttingen, Germany
* Correspondence: rdaniel@gwdg.de ; Grisebachstr. 8, 37077 Göttingen, Germany Tel.: +49 551-3933827

Content

Figure S1. Genetic organization of the insert harboring *est56* and BLAST search results for the predicted open reading frames (ORFs).
Figure S2. Multiple sequence alignment of Est56 and its homologs.
Figure S3. SDS-PAGE analysis of the purification of recombinant Est56 (including His_6-tag).
Figure S4. Substrate specificity of Est56 towards p-NP esters of different chain length
Figure S5. Unrooted phylogenetic tree of family IV esterases using neighbor-joining method.
Table S1. Features of characterized halotolerant lipolytic enzymes.
Table S2. Lipolytic enzymes derived from halophilic archaea adapting the "salt in" strategy.
Table S3. Characterized halophilic enzymes from other studies.
Table S4. Purification of recombinant Est56.
Table S5. Effect of metal ions and EDTA on Est56 activity.
Table S6. Effect of detergents on Est56 activity.
Table S7. Effect of inhibitors on Est56 activity.
Table S8. Amino acid composition comparison between halotolerant and halophilic enzymes.

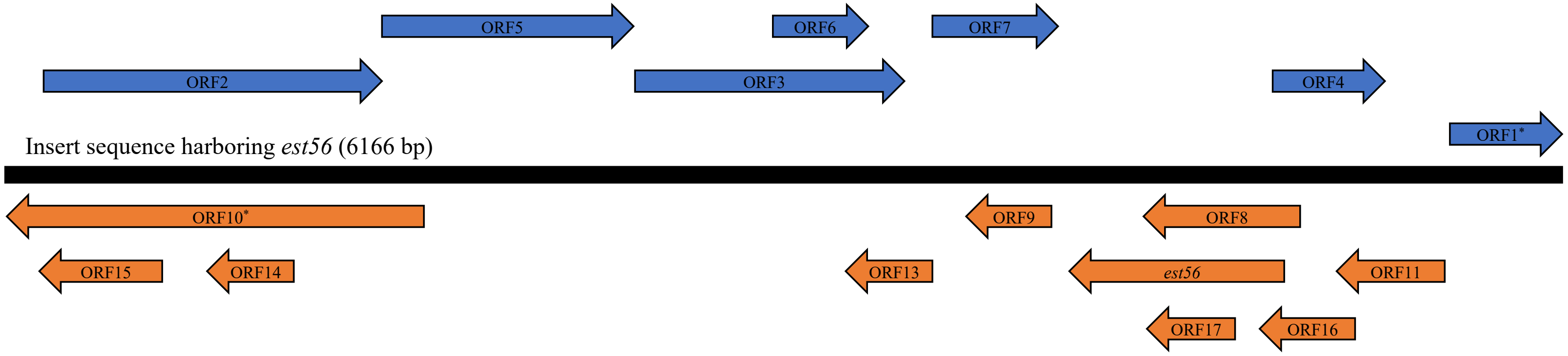

ORF	Location/Strand (+)	Putative product
ORF1	5725-6165	Hypothetical protein [Chloroflexi bacterium]
ORF2	140-1480	Homoserine dehydrogenase [Tepidiforma bonchosmolovskayae]
ORF3	2483-3559	Threonine synthase [Dehalococcoidia bacterium]
ORF4	5027-5476	No BLAST hit found
ORF5	1482-2486	Hypothetical protein [Chloroflexi bacterium]
ORF6	3036-3422	Pyridoxal-phosphate dependent enzyme [Streptomyces sp. SID7803]
ORF7	3675-4178	Hypothetical protein [Actinobacteria bacterium]

ORF	Location/Strand (-)	Putative product
ORF8	5137-4505	No BLAST hit found
ORF9	4138-3803	No BLAST hit found
ORF10	1654-2	Hypothetical protein [Candidatus Accumulibacter sp. BA-91]
ORF11	5706-5266	No BLAST hit found
est56	5070-4207	Lipase/esterase [uncultured bacterium]
ORF13	3681-3328	No BLAST hit found
ORF14	1152-799	No BLAST hit found
ORF15	615-130	Unknown [Phascolarctobacterium sp. CAG:266]
ORF16	5339-4971	No BLAST hit found
ORF17	4874-4527	No BLAST hit found

Figure S1. Genetic organization of the insert harboring *est56* and BLAST search results for the predicted open reading frames (ORFs). Only ORFs encoding putative proteins with an amino acid sequence length ≥100 are mentioned. The ORF prediction was conducted with ORFfinder (https://www.ncbi.nlm.nih.gov/orffinder/). The closest hit for each ORF was determined from the deduced protein sequences using BLASTP. * Partial ORFs.

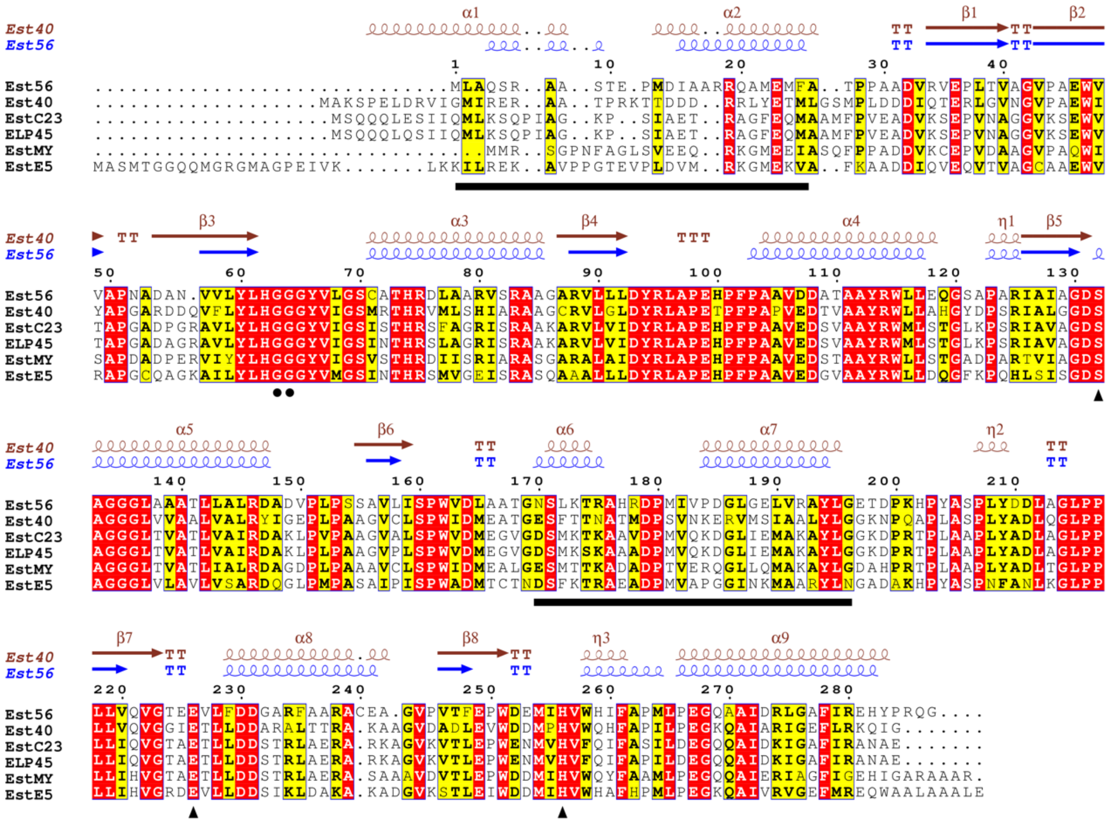

Figure S2. Multiple sequence alignment of Est56 and its homologs. Partially conserved residues are in frames. Identical residues are shaded. Triangles underneath residues indicate the catalytic triad and circles represent residues involved in the oxyanion hole. The secondary structures of Est56 and its structural analog Est40 (Li et al. 2015) are presented as: squiggles for α helices, arrows for β strands, by TT letters for turns, and η letters for 3_{10}-helices. The square bar represents regions of the cap domain. The reference esterases EstC23 (Jin et al. 2012), ELP45 (Lee et al. 2004), EstMY (Li et al. 2010) and EstE5 (Nam et al. 2009) were derived from GenBank.

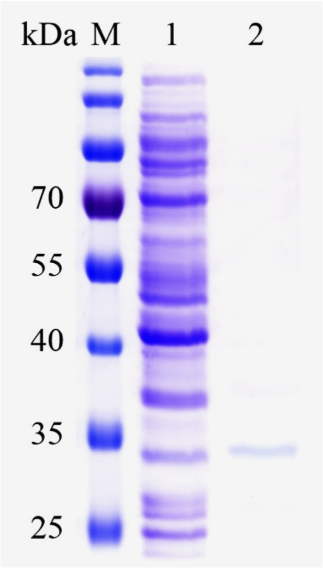

Figure S3. SDS-PAGE analysis of purification of recombinant Est56 (including His_6-tag). Lane M, standard molecular weight marker; Lane 1, cell lysate (21.5 μg); Lane 2, purified Est56 (1.0 μg).

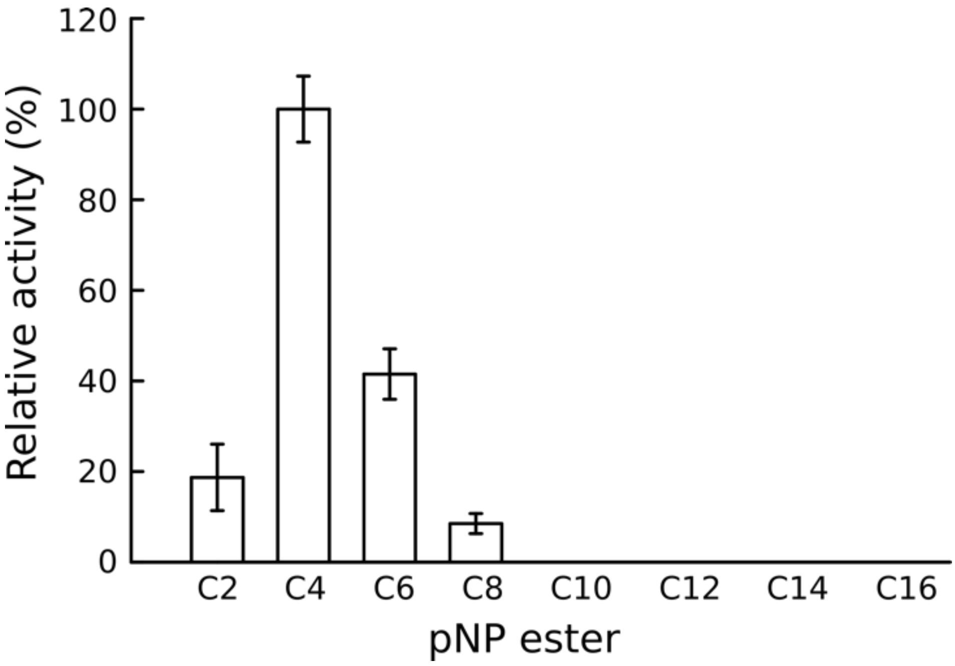

Figure S4. Substrate specificity of Est56 towards p-NP esters of different chain length. The maximal activity (189.5 U/mg) measured with p-NP butyrate (C4) was taken as 100%.

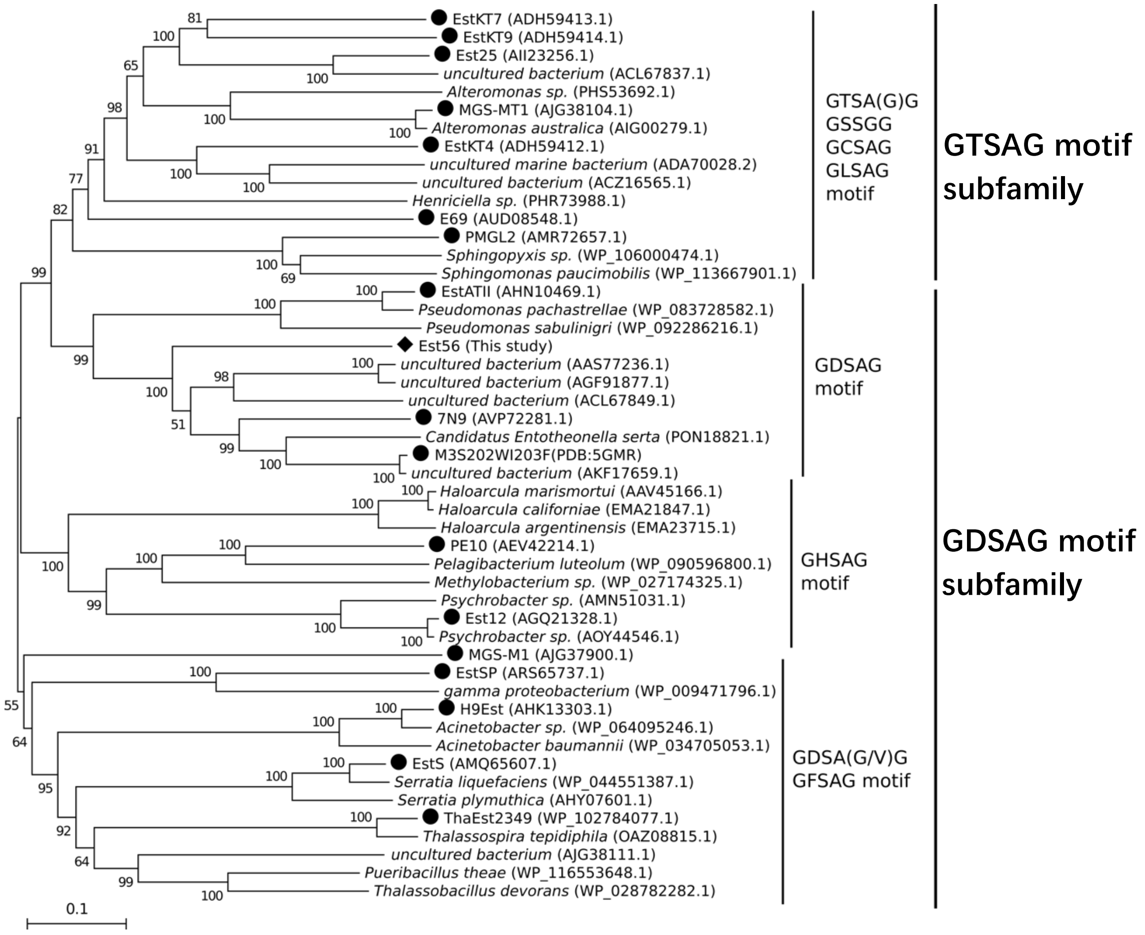

Figure S5. Unrooted phylogenetic tree of family IV esterases using neighbor-joining method. Est56 (closed diamond) and characterized halotolerant (closed circles) are depicted. With the exception of Est56, other sequences were retrieved from GenBank, with accession numbers in parentheses. Only bootstrap values greater than 50% are shown. Scale represents the number of amino acid substitutions per site.

Table S1. Features of characterized halotolerant lipolytic enzymes (HT_LIP)

Lipolytic enzyme	Organism	pI	pH_{opt}	T_{opt} (°C)	Salinity					Reference
					Effect on enzyme activity			Effect on enzyme stability		
					Salt range	Maximum activity (%)[a]	Minimum activity (%)[a]	Incubation condition	Residual activity (%)[b]	
7N9	Uncultured bacterium	4.59	8	0-30	0-24 %	100 % at 0 %	~ 55 % at 24 %	ND[d]	ND[d]	[6]
Lpc53E1	Uncultured bacterium	4.61	7	40	0-4 M	234 % at 5 M	100 % at 0 M	4 °C, 24 h	~ 100 %, over 0-5 M	[7]
EM3L4	Uncultured bacterium	4.61	7.5	35	0-4 M	ND[d]	ND[d]	35 °C, 30 min	> 100 %, over 0-4 M	[8]
PE10	*Pelagibacterium halotolerans*	4.65	7.5	45	0-4 M	~ 160 % at 3 M	100 % at 0 M	ND[d]	ND[d]	[9]
EstSP	Uncultured bacterium	4.65	8	40	0-5 M	155 % at 1 M	~ 10 % at 5 M	25 °C, 24 h	> 70 %, over 0-5 M	[10]
ABO_1251	*Alcanivorax borkumensis*	4.74	ND[d]	35	0-3.5 M	100 % at 0 M	~ 40 % at 3.5 M NaCl/KCl	ND[d]	ND[d]	[11]
E69	*Erythrobacter seohaensis*	4.76	10.5	60	0-3 M	~ 150 % at 0.5 M	~ 40 % at 3 M	ND[d]	ND[d]	[12]
MGS-K1	Uncultured bacterium	4.89	7	30	0-4 M	0.8 M NaCl[c]	ND[d]	ND[d]	ND[d]	[13]
estHIJ	*Bacillus halodurans*	4.90	7	28	0-4 M	100% at 0 M	~ 70 % at 4 M	25 °C, 6 h	~ 100 % at 4 M	[14]
EaEST	*Exiguobacterium antarcticum*	4.91	8	40	0-5 M	ND[d]	ND[d]	25 °C, 1 h	~ 100 %, over 0-5 M	[15]
Esth	*Shewanella*	4.93	8	30	0-5 M	100 % at 0 M	~ 50 % at 5 M	4 °C, 24 h	~ 100 %, over 0-5 M	[16]
ABO_1197	*Alcanivorax borkumensis*	4.93	ND[d]	30	0-3.5 M	100 % at 0 M	~ 30 % at 3.5 M NaCl/KCl	ND[d]	ND[d]	[11]
ThaEst2349	*Thalassospira* sp.	4.94	8.5	45	0-4 M	283 % at 3 M	40 % at 4 M	4 °C, 24 h	>280 %, over 1-3 M	[17]
Est56	Uncultured bacterium	4.97	8	50	0-4 M	~ 140 % at 1.5 M NaCl or 1 M KCl	~ 40 % at 4M NaCl/KCl	10 °C, 24 h	~ 100 %, over 0-4 M NaCl/KCl	**This study**
Lip3	Uncultured bacterium	4.98	8	35	0-4 M	675 % at 3 M NaCl	100 % at 0 M	4 °C, 24 h	> 100 %, over 0-3 M	[18]
EstS	*Serratia* sp.	5.05	8.5	10	0-4 M	100 % at 0 M	94 % at 4 M	4 °C, 24 h	> 80 %, over 0-4 M	[19]
MGS-RG1	Uncultured bacterium	5.07	8	45	0-4 M	~ 250 % at 3.2 M	100 % at 0 M	ND[d]	ND[d]	[20]
E25	Uncultured bacterium	5.16	8.5	50	0-4 M	~ 130 % at 1 M	~ 50 % at 4 M	ND[d]	ND[d]	[21]
M3S202WI 203F	Uncultured bacterium	5.17	ND[d]	55	0-4 M	~ 110 % at 0.5 M	~ 60 % at 4 M	20 °C, 1 h	> 70 %, over 0-4 M	[22]
YbfF	*Halomonas elongata*	5.25	8	ND[d]	0-4 M	100 % at 2 M	~15 % at 0 M	ND[d]	ND[d]	[23]
EstSL3	*Alkalibacterium* sp.	5.28	9	30	0-4 M	~ 105 % at 2 M	98 % at 4 M	37 °C, 2 h	~ 100 %, over 0-4 M	[24]

MGS-RG2	Uncultured bacterium	5.31	8	50	0-4 M	~ 250 % at 3.6 M	100 % at 0 M	ND[d]	ND[d]	[20]
BlEst1	*Bacillus licheniformis*	5.33	7	40	0-5 M	100 % at 1 M	~ 70 % at 5 M	ND[d]	ND[d]	[25]
Est10	*Psychrobacter pacificensis*	5.35	7.5	25	0-5 M	143.2 % at 2 M	~ 80 % at 5 M	4 °C, 6.5 h	> 100 %, over 0-5 M	[26]
MGS0010	Uncultured bacterium	5.4	ND[d]	30	0-3.5 M	~ 250 % at 3.5 M	100 % at 0 M	ND[d]	ND[d]	[11]
BlEstA	*Bacillus licheniformis*	5.54	9	30	0-3 M	165 % at 2 M	100 % at 0 M	ND[d]	ND[d]	[27]
EstWSD	Uncultured bacterium	5.61	7	50	0-5 M	~ 140 % at 1 M	~ 50 % at 5 M	ND[d]	ND[d]	[28]
EstSHJ2	*Chromohalobacter canadensis*	5.71	8	50	0-5 M	100 % at 2.5 M	~10 % at 0 M	ND[d]	ND[d]	[29]
PMGL2	Uncultured bacterium	5.72	8.5	45	0-1.75 M	165 % at 0.25 M	84 % at 1.75 M	ND[d]	ND[d]	[30]
MGS-M1	Uncultured bacterium	5.77	8	25	0-4 M	3.6 M NaCl[c]	ND[d]	ND[d]	ND[d]	[13]
EstKT4	Uncultured bacterium	5.81	8.5	40	0-4 M	ND[d]	ND[d]	35 °C, 30 min	> 50%, over 0-3.5 M	[31]
EstKT7	Uncultured bacterium	5.84	8	35	0-4 M	ND[d]	ND[d]	35 °C, 30 min	> 50%, over 0-3 M	[31]
LipC12	Uncultured bacterium	5.98	9	30	0-4 M	1501 % at 1.5 M	100 % at 0 M	4 °C, 24 h	~ 100 %, over 0-3.7 M	[32]
EstKT9	Uncultured bacterium	6.1	8.5	45	0-4 M	ND[d]	ND[d]	35 °C, 30 min	> 50%, over 0-3.5 M	[31]
lp_3505	*Lactobacillus plantarum*	6.12	6	5	0-25 %	~ 250 % at 5 %	~ 70 % at 25 %	ND[d]	ND[d]	[33]
Est9x	Uncultured bacterium	6.17	8	65	0-4 M	~ 190 % at 4 M	100 % at 0 M	ND[d]	ND[d]	[34]
Est12	*Psychrobacter celer*	6.5	7.5	35	0-4.5 M	ND[d]	ND[d]	25 °C, 12 h	~ 100 %, over 0-4.5 M	[35]
EstATII	Uncultured bacterium	7.11	8.5	65	0-4 M	ND[d]	~ 50 % at 4 M	ND[d]	ND[d]	[36]
Est-OKK	Uncultured bacterium	7.82	9	50	0-3 M	~ 130 % at 1.5 M	~ 100 % at 3 M	RT, 4 h	~ 100 %, over 0-3 M	[37]
LipJ2	*Janibacter* sp.	8.25	9	80	1, 10 mM	~ 250 % at 0.1 mM NaCl	100 % at 0 M	ND[d]	ND[d]	[38]
MGS-MT1	Uncultured bacterium	8.4	8.5	50	0-4 M		ND[d]	ND[d]	ND[d]	[13]
EstLiu	*Zunongwangia profunda*	8.42	8	30	0-4.5 M	100 % at 0 M	57 % at 4.5 M	4 °C, 12 h	> 80 %, over 0-4.5 M	[39]
H9Est	Uncultured bacterium	8.72	8	40	0-2.5 M	~ 150 % at 1 M	~ 50 % at 2.5 M	ND[d]	ND[d]	[40]
H8	Uncultured bacterium	9.09	10	35	0-5 M	~ 105 % at 4 M	~ 10 % at 5 M	0 °C, 1 h	> 80 %, over 0-4.7 M	[41]
Est700	*Bacillus licheniformis*	9.44	8	30	0-5 M	588 % at 3.5 M	100 % at 0 M	4 °C, 1 h	~ 100 %, over 0-5 M	[42]
BmEST	*Bacillus mojavensis*	ND[d]	8	80	0-25 %	~ 300 % at 20 %	100 % at 0 %	80 °C, 1 h	> 60 %, over 0-25 %	[43]

[a] The activity measured without salt (NaCl, if not mentioned) was taken as 100 %

[b] The activity measured after incubating in salt-free (NaCl, unless stated otherwise) buffer was defined as 100%

[c] The activity at $Salt_{opt}$ was set as 100 %

[d] No data

Table S2. Lipolytic enzymes (HP_Lip) derived from halophilic archaea adapting the "salt in" strategy

Lipolytic enzyme	Length (aa)	pI	Microorganism	GeneBank/PDB Accession Nr.	Family	Halophile	Growth condition (NaCl, %)
HP_Lip_1	285	4.45	*Halococcus thailandensis*	EMA51434.1	*Halobacteriaceae*	Extreme	20% - 30%
HP_Lip_2	292	4.55	*Halococcus morrhuae*	EMA45705.1	*Halobacteriaceae*	Extreme	>12%, 23.3%
HP_Lip_3	320	4.16	*Halococcus saccharolyticus*	EMA45019.1	*Halobacteriaceae*	Extreme	15% - satr, optimum 25%
HP_Lip_4	285	4.5	*Halococcus hamelinensis*	EMA39292.1	*Halobacteriaceae*	Moderate	12.5% - 30%, optimum 15%
HP_Lip_5	263	4.4	*Halosimplex carlsbadense*	ELZ28160.1	*Halobacteriaceae*	Extreme	20% - 30% (optimum 25%)
HP_Lip_6	261	4.54	*Halosimplex carlsbadense*	ELZ24957.1	*Halobacteriaceae*	Extreme	20% - 30% (optimum 25%)
HP_Lip_7	330	4.17	*Haladaptatus sp.*	KZN24148.1	*Halobacteriaceae*	Extreme	ND[b]
HP_Lip_8	333	4.32	*Halorhabdus utahensis*	ACV11819.1	*Halobacteriaceae*	Extreme	9% - 30%, optimum 27%
HP_Lip_9	340	4.34	*Halorhabdus utahensis*	ACV10409.1	*Halobacteriaceae*	Extreme	9% - 30%, optimum 27%
HP_Lip_10	258	4.57	*Haloarcula marismortui*	AAV45777.1	*Halobacteriaceae*	Extreme	optimum 20% - 23%
HP_Lip_11	318	4.22	*Haloarcula hispanica*	AHB65276.1	*Halobacteriaceae*	Moderate	>12%
HP_Lip_12	377	4.32	*Haloarcula japonica*	WP_004591147.1	*Halobacteriaceae*	Extreme	15% - 30%, optimum 20%
HP_Lip_13	318	4.18	*Haloarcula japonica*	EMA29911.1	*Halobacteriaceae*	Extreme	15% - 30%, optimum 20%
HP_Lip_14	318	4.19	*Haloarcula vallismortis*	EMA07756.1	*Halobacteriaceae*	Extreme	>15%, optimum 25%
HP_Lip_15	376	4.3	*Haloarcula vallismortis*	WP_004515030.1	*Halobacteriaceae*	Extreme	>15%, optimum 25%
HP_Lip_16	314	4.23	*Haloprofundus marisrubri*	KTG11548.1	*Haloferacaceae*	Extreme	min 7%
HP_Lip_17	260	4.35	*Haloprofundus marisrubri*	KTG08679.1	*Haloferacaceae*	Extreme	min 7%
HP_Lip_18	217	4.44	*Halogeometricum pallidum*	ELZ32922.1	*Haloferacaceae*	Moderate	optimum 18.1%
HP_Lip_19	275	4.48	*Halogeometricum pallidum*	ELZ27142.1	*Haloferacaceae*	Moderate	optimum 18.1%
HP_Lip_20	215	4.53	*Halogeometricum borinquense*	ELY30686.1	*Haloferacaceae*	Extreme	min 8%, optimum 20% -
HP_Lip_21	344	4.27	*Natronolimnobius baerhuensis*	OVE85190.1	*Natrialbaceae*	Extreme	optimum 20%
HP_Lip_22	455	4.54	*Haloterrigena mahii*	OAQ52820.1	*Natrialbaceae*	Extreme	optimum 20.4% - 29.2%

Table S3. Characterized halophilic enzymes (HP_Enz) from other studies

Protein	Length (aa)	pI	Organism	Domain	Family	Halophile	Growth condition (NaCl, %)	Reference
Malate dehydrogenase	303	4.2	*Haloarcula marismortui*	Archaea	*Halobacteriaceae*	Extreme	10% - 30%, optimum 20% - 23%	[44]
Malate dehydrogenase	304	4.2	*Haloarcula marismortui*	Archaea	*Halobacteriaceae*	Extreme	10% - 30%, optimum 20% - 23%	[45]
Catalase-peroxidase	731	4.32	*Haloarcula marismortui*	Archaea	*Halobacteriaceae*	Extreme	10% - 30%, optimum 20% - 23%	[46]
Esterase	327	4.24	*Haloarcula marismortui*	Archaea	*Halobacteriaceae*	Extreme	10% - 30%, optimum 20% - 23%	[47]
Nucleoside diphosphate kinase	161	4.37	*Halobacterium salinarum*	Archaea	*Halobacteriaceae*	Moderate	>12%, NaCl saturation	[48]
Nucleoside diphosphate kinase	164	4.42	*Halobacterium salinarum*	Archaea	*Halobacteriaceae*	Moderate	>12%, NaCl saturation	[48]
RNase H1	199	4.36	*Halobacterium salinarum*	Archaea	*Halobacteriaceae*	Moderate	>12%, NaCl saturation	[49]
Phosphatase	431	4.35	*Halobacterium salinarum*	Archaea	*Halobacteriaceae*	Moderate	>12%, NaCl saturation	[50]
Dehydrogenase	435	4.39	*Halobacterium salinarum*	Archaea	*Halobacteriaceae*	Moderate	>12%, NaCl saturation	[51]
DNA protecting protein	182	4.3	*Halobacterium salinarum*	Archaea	*Halobacteriaceae*	Moderate	>12%, NaCl saturation	[52]
Beta-galactosidase	663	4.54	*Haloferax lucentense*	Archaea	*Haloferacaceae*	Extreme	10.5% - 29.8% (optimum 25.1%)	[53]
DNA ligase	699	4.34	*Haloferax volcanii*	Archaea	*Haloferacaceae*	Extreme	6% - 29%, optimum 10% - 15%	[54]
Dihydrofolate reductase	162	4.45	*Haloferax volcanii*	Archaea	*Haloferacaceae*	Extreme	6% - 29%, optimum 10% - 15%	[55]
Glucose dehydrogenase	357	4.55	*Haloferax mediterranei*	Archaea	*Haloferacaceae*	Moderate	>12%	[56]
Alpha-amylase	504	4.11	*Natronococcus sp.*	Archaea	*Natrialbaceae*	Moderate	>12%	[57]
Esterase	316	4.38	unclutured bacterium	Bacterium	ND[b]	ND[b]	ND[b]	[13]

[a] no data

Table S4. Purification of recombinant Est56.

Purification step	Total activity (U)	Total protein (mg)	Specific activity (U/mg)	Purification (fold)	Yield (%)
Crude extract	239.8	193.7	1.24	1.0	100
Ni-TED	108.8	1.2	90.44	73.0	45.4

Table S5. Effect of metal ions and EDTA on Est56 activity

Metal ions	Concentration (mM)	Relative activity (%) [a]
Al^{3+}	1	127.5±3.0
Al^{3+}	10	142.8±6.3
Ca^{2+}	1	126.5±10.3
Ca^{2+}	10	136.9±9.3
Mg^{2+}	1	106.1±0.9
Mg^{2+}	10	105.5±5.6
Mn^{2+}	1	113.2±2.5
Mn^{2+}	10	82.4±3.5
Zn^{2+}	1	75.4±8.2
$Zn2^{+}$	10	83.6±7.3
Fe^{2+}	1	82.4±6.6
Fe^{2+}	10	13.2±1.9
Fe^{3+}	1	60.9±5.0
Fe^{3+}	10	19.5±6.3
Ni^{2+}	1	13.3±4.1
Ni^{2+}	10	22.0±4.4
Cu^{2+}	1	16.7±8.7
Cu^{2+}	10	23.2±2.2
EDTA	1	119.4±2.5
EDTA	10	93.8±10.5

[a] The effects of metal ions on Est56 activity were measured under standard assay conditions. Specific activity (93.1 U/mg) assayed without additive was taken as 100%.

[b] Not detectable.

Table S6. Effect of detergents on Est56 activity

Detergent	Concentration (v/v, %)	Relative activity (%) [a]
Triton X-100	0.1	156.8±3.4
Triton X-100	1	44.6±2.8
Triton X-100	5	18.3±3.6
Tween 20	0.1	154.3±2.3
Tween 20	1	53.6±3.0
Tween 20	5	ND[b]
Tween 80	0.1	112.7±1.7
Tween 80	1	36.6±1.8
Tween 80	5	11.4±1.6

[a] The effects of detergents on Est56 activity were measured under standard assay conditions. Specific activity (93.1 U/mg) assayed without additive was taken as 100%.

[b] Not detectable.

Table S7. Effect of inhibitors on Est56 activity

Inhibitor	Concentration (mM)	Relative activity (%) [a]
DTT	1	102.2±9.3
DTT	10	46.0±1.4
PMSF	1	69.9±0.7
PMSF	10	10.1±1.2
DEPC	1	1.9±1.1
DEPC	10	ND[b]

[a] The effects of inhibitors on Est56 activity were measured under standard assay conditions. Specific activity (93.1 U/mg) assayed without additive was taken as 100%.

[b] Not detectable.

Table S8. Amino acid composition comparison between halotolerant and halophilic enzymes

Amino acid	HT (%)[a]	HP_Lip (%)[a]	HP_Enz (%)[a]	SIMPER analysis[b]		
				HT vs HP_Lip Contribution (%)[c]	HT vs HP_Enz Contribution (%)[d]	HP_Lip vs HP_Enz Contribution (%)[e]
Asp (D)	5.92±1.39	9.87±1.39	10.47±1.99	10.44 (1)	11.58 (1)	6.32 (5)
Ala (A)	10.68±3.19	12.55±2.07	11.36±2.79	8.76 (2)	8.40 (3)	8.73 (1)
Lys (K)	3.74±2.30	1.01±0.48	2.16±1.26	7.34 (3)	5.77 (5)	4.24 (14)
Glu (E)	5.27±1.47	7.86±1.59	8.87±1.98	7.13 (4)	9.26 (2)	6.97 (3)
Ile (I)	5.14±1.97	2.80±1.16	3.86±1.16	6.75 (5)	4.92 (8)	5.20 (9)
Gly (G)	8.42±1.80	9.22±1.89	8.68±1.80	5.59 (6)	5.04 (7)	6.86 (4)
Leu (L)	9.82±1.92	8.30±1.25	7.57±1.19	5.57 (7)	6.59 (4)	4.87 (10)
Asn (N)	3.51±1.56	1.66±0.94	2.49±1.07	5.41 (8)	4.12 (13)	4.34 (12)
Thr (T)	4.93±1.61	5.41±1.61	5.56±1.50	4.71 (9)	4.3 (12)	5.39 (6)
Arg (R)	4.73±1.72	5.60±1.09	5.70±1.31	4.68 (10)	4.67 (11)	4.27 (13)
Pro (P)	5.98±1.50	6.11±1.70	4.83±1.93	4.59 (11)	5.31 (6)	7.393 (2)
Ser (S)	5.93±1.68	5.21±1.33	4.88±1.62	4.39 (12)	4.77 (10)	5.371 (7)
Val (V)	7.52±1.64	7.90±1.13	8.07±1.71	4.2 (13)	4.83 (9)	5.27 (8)
Gln (Q)	3.57±1.29	2.55±0.94	2.79±0.85	3.72 (14)	3.28 (17)	3.34 (17)
Met (M)	2.88±0.83	1.54±0.73	1.86±1.00	3.72 (15)	3.36 (16)	3.26 (18)
His (H)	2.70±1.10	3.58±0.79	2.94±1.45	3.37 (16)	3.51 (14)	4.80 (11)
Phe (F)	3.61±1.21	3.65±0.81	3.13±1.20	3.03 (17)	3.45 (15)	3.94 (15)
Tyr (Y)	3.34±0.98	3.24±1.05	2.91±0.87	2.9 (18)	2.73 (18)	3.47 (16)
Trp (W)	1.54±0.76	1.40±0.63	1.46±0.99	2.02 (19)	2.46 (19)	2.99 (19)
Cys (C)	0.76±0.65	0.72±0.51	0.41±0.47	1.68 (20)	1.65 (20)	1.99 (20)

[a] Data shown are averages with the standard deviation per group.

[b] SIMPER analysis the contribution of each amino acid to the dissimilarity between groups, with permutations 9999. Data shown are contributions (%) with the rank in the brackets.

[c] SIMPER-revealed average dissimilarity was 19.38.

[d] SIMPER-revealed average dissimilarity was 20.03.

[e] SIMPER-revealed average dissimilarity was 15.31.

References

1. Li, P.; Chen, X.; Ji, P.; Li, C.; Wang, P.; Zhang, Y.; Xie, B.; Qin, Q.; Su, H.; Zhou, B.; et al. Interdomain hydrophobic interactions modulate the thermostability of microbial esterases from the hormone-sensitive lipase family. *J. Biol. Chem.* **2015**, *290*, 11188–11198, doi:10.1074/jbc.M115.646182.
2. Jin, P.; Pei, X.; Du, P.; Yin, X.; Xiong, X.; Wu, H.; Zhou, X.; Wang, Q. Overexpression and characterization of a new organic solvent-tolerant esterase derived from soil metagenomic DNA. *Bioresour. Technol.* **2012**, *116*, 234–240, doi:10.1016/j.biortech.2011.10.087.
3. Lee, S.W.; Won, K.; Lim, H.K.; Kim, J.C.; Choi, G.J.; Cho, K.Y. Screening for novel lipolytic enzymes from uncultured soil microorganisms. *Appl. Microbiol. Biotechnol.* **2004**, *65*, 720–726, doi:10.1007/s00253-004-1722-3.
4. Li, J.; Zhang, K.; Han, W. Cloning and biochemical characterization of a novel lipolytic gene from activated sludge metagenome, and its gene product. *Microb. Cell Fact.* **2010**, *9*, 83, doi:10.1186/1475-2859-9-83.
5. Nam, K.H.; Kim, M.Y.; Kim, S.J.; Priyadarshi, A.; Lee, W.H.; Hwang, K.Y. Structural and functional analysis of a novel EstE5 belonging to the subfamily of hormone-sensitive lipase. *Biochem. Biophys. Res. Commun.* **2009**, *379*, 553–556, doi:10.1016/j.bbrc.2008.12.085.
6. Borchert, E.; Selvin, J.; Kiran, S.G.; Jackson, S.A.; O'Gara, F.; Dobson, A.D.W. A novel cold active esterase from a deep sea sponge *Stelletta normani* metagenomic library. *Front. Mar. Sci.* **2017**, *4*, 1–13, doi:10.3389/fmars.2017.00287.
7. Selvin, J.; Kennedy, J.; Lejon, D.P.H.; Kiran, G.S.; Dobson, A.D.W. Isolation identification and biochemical characterization of a novel halo-tolerant lipase from the metagenome of the marine sponge *Haliclona simulans*. *Microb. Cell Fact.* **2012**, *11*, 72, doi:10.1186/1475-2859-11-72.
8. Lee, J.H.; Jeon, J.H.; Kim, J.T.; Lee, H.S.; Kim, S.J.; Kang, S.G.; Choi, S.H. Novel lipolytic enzymes identified from metagenomic library of deep-sea sediment. *Evidence-based Complement. Altern. Med.* **2011**, *2011*, doi:10.1155/2011/271419.
9. Jiang, X.; Huo, Y.; Cheng, H.; Zhang, X.; Zhu, X.; Wu, M. Cloning, expression and characterization of a halotolerant esterase from a marine bacterium *Pelagibacterium halotolerans* B2T. *Extremophiles* **2012**, *16*, 427–435, doi:10.1007/s00792-012-0442-3.
10. Jayanath, G.; Mohandas, S.P.; Kachiprath, B.; Solomon, S.; Sajeevan, T.P.; Bright Singh, I.S.; Philip, R. A novel solvent tolerant esterase of GDSGG motif subfamily from solar saltern through metagenomic approach: Recombinant expression and characterization. *Int. J. Biol. Macromol.* **2018**, *119*, 393–401, doi:10.1016/j.ijbiomac.2018.06.057.
11. Tchigvintsev, A.; Tran, H.; Popovic, A.; Kovacic, F.; Brown, G.; Flick, R.; Hajighasemi, M.; Egorova, O.; Somody, J.C.; Tchigvintsev, D.; et al. The environment shapes microbial enzymes: five cold-active and salt-resistant carboxylesterases from marine metagenomes. *Appl. Microbiol. Biotechnol.* **2015**, *99*, 2165–2178, doi:10.1007/s00253-014-6038-3.
12. Huo, Y.Y.; Rong, Z.; Jian, S.L.; Xu, C. Di; Li, J. xi; Xu, X.W. A novel halotolerant thermoalkaliphilic esterase from marine bacterium *Erythrobacter seohaensis* SW-135. *Front. Microbiol.* **2017**, *8*, 2315, doi:10.3389/fmicb.2017.02315.
13. Alcaide, M.; Stogios, P.J.; Lafraya, Á.; Tchigvintsev, A.; Flick, R.; Bargiela, R.; Chernikova, T.N.; Reva, O.N.; Hai, T.; Leggewie, C.C.; et al. Pressure adaptation is linked to thermal adaptation in salt-saturated marine habitats. *Environ. Microbiol.* **2015**, *17*, 332–345, doi:10.1111/1462-2920.12660.
14. Noby, N.; Hussein, A.; Saeed, H.; Embaby, A.M. Recombinant cold-adapted halotolerant, organic solvent-stable esterase (estHIJ) from *Bacillus halodurans*. *Anal. Biochem.* **2020**, *591*, 113554, doi:10.1016/j.ab.2019.113554.
15. Lee, C.W.; Kwon, S.; Park, S.H.; Kim, B.Y.; Yoo, W.; Ryu, B.H.; Kim, H.W.; Shin, S.C.; Kim, S.; Park, H.; et al. Crystal structure and functional characterization of an esterase (EaEST) from *Exiguobacterium antarcticum*. *PLoS One* **2017**, *12*, e0169540, doi:10.1371/journal.pone.0169540.
16. Hang, Y.; Ran, S.; Wang, X.; Jiao, J.; Wang, S.; Liu, Z. Mutational analysis and stability characterization of a novel esterase of lipolytic enzyme family VI from *Shewanella* sp. *Int. J. Biol. Macromol.* **2016**, *93*, 655–664, doi:10.1016/j.ijbiomac.2016.09.032.
17. De Santi, C.; Leiros, H.K.S.; Di Scala, A.; de Pascale, D.; Altermark, B.; Willassen, N.P. Biochemical characterization and structural analysis of a new cold-active and salt-tolerant esterase from the marine bacterium *Thalassospira* sp. *Extremophiles* **2016**, *20*, 323–336, doi:10.1007/s00792-016-0824-z.

18. De Santi, C.; Altermark, B.; Pierechod, M.M.; Ambrosino, L.; de Pascale, D.; Willassen, N.-P. Characterization of a cold-active and salt tolerant esterase identified by functional screening of Arctic metagenomic libraries. *BMC Biochem.* **2016**, *17*, doi:10.1186/s12858-016-0057-x.
19. Jiang, H.; Zhang, S.; Gao, H.; Hu, N. Characterization of a cold-active esterase from *Serratia* sp. and improvement of thermostability by directed evolution. *BMC Biotechnol.* **2016**, *16*, doi:10.1186/s12896-016-0235-3.
20. Alcaide, M.; Tchigvintsev, A.; Martínez-Martínez, M.; Popovic, A.; Reva, O.N.; Lafraya, Á.; Bargiela, R.; Nechitaylo, T.Y.; Matesanz, R.; Cambon-Bonavita, M.-A.; et al. Identification and characterization of carboxyl esterases of gill chamber-associated microbiota in the deep-sea shrimp *Rimicaris exoculata* by using functional metagenomics. *Appl. Environ. Microbiol.* **2015**, *81*, 2125–2136, doi:10.1128/AEM.03387-14.
21. Li, P.Y.; Ji, P.; Li, C.Y.; Zhang, Y.; Wang, G.L.; Zhang, X.Y.; Xie, B. Bin; Qin, Q.L.; Chen, X.L.; Zhou, B.C.; et al. Structural basis for dimerization and catalysis of a novel sterase from the GTSAG motif subfamily of the bacterial hormone-sensitive lipase family. *J. Biol. Chem.* **2014**, *289*, 19031–19041, doi:10.1074/jbc.M114.574913.
22. Li, P.-Y.; Zhang, Y.; Xie, B.-B.; Zhang, Y.-Q.; Hao, J.; Wang, Y.; Wang, P.; Li, C.-Y.; Qin, Q.-L.; Zhang, X.-Y.; et al. Structural and mechanistic insights into the improvement of the halotolerance of a marine microbial esterase by increasing intra- and interdomain hydrophobic interactions. *Appl. Environ. Microbiol.* **2017**, *83*, e01286-17, doi:10.1128/AEM.01286-17.
23. Yoo, W.; Kim, B.; Jeon, S.; Kim, K.K.; Kim, T.D. Identification, characterization, and immobilization of a novel YbfF esterase from *Halomonas elongata*. *Int. J. Biol. Macromol.* **2020**, *165*, 1139–1148, doi:10.1016/j.ijbiomac.2020.09.247.
24. Wang, G.; Wang, Q.; Lin, X.; Ng, T.B.; Yan, R.; Lin, J.; Ye, X. A novel cold-adapted and highly salt-tolerant esterase from *Alkalibacterium* sp. SL3 from the sediment of a soda lake. *Sci. Rep.* **2016**, *6*, 19494, doi:10.1038/srep19494.
25. Nakamura, A.M.; Kadowaki, M.A.S.; Godoy, A.; Nascimento, A.S.; Polikarpov, I. Low-resolution envelope, biophysical analysis and biochemical characterization of a short-chain specific and halotolerant carboxylesterase from *Bacillus licheniformis*. *Int. J. Biol. Macromol.* **2018**, *120*, 1893–1905, doi:10.1016/j.ijbiomac.2018.10.003.
26. Wu, G.; Wu, G.; Zhan, T.; Shao, Z.; Liu, Z. Characterization of a cold-adapted and salt-tolerant esterase from a psychrotrophic bacterium *Psychrobacter pacificensis*. *Extremophiles* **2013**, *17*, 809–819, doi:10.1007/s00792-013-0562-4.
27. Leite, A.E.T.; Briganti, L.; de Araújo, E.A.; Pellegrini, V. de O.A.; Camilo, C.M.; Polikarpov, I. Low-resolution molecular shape, biochemical characterization and emulsification properties of a halotolerant esterase from *Bacillus licheniformis*. *Eur. Biophys. J.* **2020**, *49*, 435–447, doi:10.1007/s00249-020-01448-7.
28. Wang, S.; Wang, K.; Li, L.; Liu, Y. Isolation and characterization of a novel organic solvent-tolerant and halotolerant esterase from a soil metagenomic library. *J. Mol. Catal. B. Enzym.* **2013**, *95*, 1–8, doi:10.1016/j.molcatb.2013.05.015.
29. Wang, M.; Ai, L.; Zhang, M.; Wang, F.; Wang, C. Characterization of a novel halotolerant esterase from *Chromohalobacter canadensis* isolated from salt well mine. *3 Biotech* **2020**, *10*, 430, doi:10.1007/s13205-020-02420-0.
30. Petrovskaya, L.E.; Novototskaya-Vlasova, K.A.; Spirina, E. V.; Durdenko, E. V.; Lomakina, G.Y.; Zavialova, M.G.; Nikolaev, E.N.; Rivkina, E.M. Expression and characterization of a new esterase with GCSAG motif from a permafrost metagenomic library. *FEMS Microbiol. Ecol.* **2016**, *92*, fiw046, doi:10.1093/femsec/fiw046.
31. Jeon, J.H.; Lee, H.S.; Kim, J.T.; Kim, S.J.; Choi, S.H.; Kang, S.G.; Lee, J.H. Identification of a new subfamily of salt-tolerant esterases from a metagenomic library of tidal flat sediment. *Appl. Microbiol. Biotechnol.* **2012**, *93*, 623–631, doi:10.1007/s00253-011-3433-x.
32. Glogauer, A.; Martini, V.P.; Faoro, H.; Couto, G.H.; Müller-Santos, M.; Monteiro, R.A.; Mitchell, D.A.; de Souza, E.M.; Pedrosa, F.O.; Krieger, N. Identification and characterization of a new true lipase isolated through metagenomic approach. *Microb. Cell Fact.* **2011**, *10*, 54, doi:10.1186/1475-2859-10-54.
33. Esteban-Torres, M.; Santamaría, L.; de las Rivas, B.; Muñoz, R. Characterisation of a cold-active and salt-tolerant esterase from *Lactobacillus plantarum* with potential application during cheese ripening. *Int. Dairy J.* **2014**, *39*, 312–315, doi:10.1016/j.idairyj.2014.08.004.
34. Fang, Z.; Li, J.; Wang, Q.; Fang, W.; Peng, H.; Zhang, X.; Xiao, Y. A novel esterase from a marine metagenomic library exhibiting salt tolerance ability. *J. Microbiol. Biotechnol.* **2014**, *24*, 771–780, doi:10.4014/jmb.1311.11071.

35. Wu, G.; Zhang, S.; Zhang, H.; Zhang, S.; Liu, Z. A novel esterase from a psychrotrophic bacterium *Psychrobacter celer* 3Pb1 showed cold-adaptation and salt-tolerance. *J. Mol. Catal. B Enzym.* **2013**, *98*, 119–126, doi:10.1016/j.molcatb.2013.10.012.
36. Mohamed, Y.M.; Ghazy, M.A.; Sayed, A.; Ouf, A.; El-Dorry, H.; Siam, R. Isolation and characterization of a heavy metal-resistant, thermophilic esterase from a Red Sea Brine Pool. *Sci. Rep.* **2013**, *3*, 3358, doi:10.1038/srep03358.
37. Yang, X.; Wu, L.; Xu, Y.; Ke, C.; Hu, F.; Xiao, X.; Huang, J. Identification and characterization of a novel alkalistable and salt-tolerant esterase from the deep-sea hydrothermal vent of the East Pacific Rise. *Microbiology* **2018**, *7*, e00601, doi:10.1002/mbo3.601.
38. Castilla, A.; Panizza, P.; Rodríguez, D.; Bonino, L.; Díaz, P.; Irazoqui, G.; Rodríguez Giordano, S. A novel thermophilic and halophilic esterase from *Janibacter* sp. R02, the first member of a new lipase family (Family XVII). *Enzyme Microb. Technol.* **2017**, *98*, 86–95, doi:10.1016/J.ENZMICTEC.2016.12.010.
39. Rahman, M.A.; Culsum, U.; Tang, W.; Zhang, S.W.; Wu, G.; Liu, Z. Characterization of a novel cold active and salt tolerant esterase from *Zunongwangia profunda*. *Enzyme Microb. Technol.* **2016**, *85*, 1–11, doi:10.1016/j.enzmictec.2015.12.013.
40. Santi, C. De; Zhai, L.; Ambrosino, L.; Tedesco, P.; Zhou, C.; Xue, Y.; Ma, Y.; Pascale, D. de Identification and characterization of a novel salt-tolerant esterase from a Tibetan glacier metagenomic library. *Biotechnol. Prog.* **2015**, *31*, 890–899, doi:10.1002/btpr.
41. Zhang, Y.; Hao, J.; Zhang, Y.Q.; Chen, X.L.; Xie, B. Bin; Shi, M.; Zhou, B.C.; Zhang, Y.Z.; Li, P.Y. Identification and characterization of a novel salt-tolerant esterase from the deep-sea sediment of the South China Sea. *Front. Microbiol.* **2017**, *8*, 441, doi:10.3389/fmicb.2017.00441.
42. Zhang, W.; Xu, H.; Wu, Y.; Zeng, J.; Guo, Z.; Wang, L.; Shen, C.; Qiao, D.; Cao, Y. A new cold-adapted, alkali-stable and highly salt-tolerant esterase from *Bacillus licheniformis*. *Int. J. Biol. Macromol.* **2018**, *111*, 1183–1193, doi:10.1016/J.IJBIOMAC.2018.01.152.
43. Adıgüzel, A.O. Production and characterization of thermo-, halo- and solvent-stable esterase from *Bacillus mojavensis* TH309. *Biocatal. Biotransformation* **2020**, *38*, 210–226, doi:10.1080/10242422.2020.1715370.
44. Richard, S.B.; Madern, D.; Garcin, E.; Zaccai, G. Halophilic adaptation: novel solvent protein interactions observed in the 2.9 and 2.6 A resolution structures of the wild type and a mutant of malate dehydrogenase from *Haloarcula marismortui*. *Biochemistry* **2000**, *39*, 992–1000.
45. Fioravanti, E.; Vellieux, F.M.D.; Amara, P.; Madern, D.; Weik, M. Specific radiation damage to acidic residues and its relation to their chemical and structural environment. *J. Synchrotron Radiat.* **2007**, *14*, 84–91, doi:10.1107/S0909049506038623.
46. Yamada, Y.; Fujiwara, T.; Sato, T.; Igarashi, N.; Tanaka, N. The 2.0 A crystal structure of catalase-peroxidase from *Haloarcula marismortui*. *Nat. Struct. Biol.* **2002**, *9*, 691–695, doi:10.1038/nsb834.
47. Rao, L.; Zhao, X.; Li, Y.; Xue, Y.; Ma, Y.; Lu, J.R. Solution behavior and activity of a halophilic esterase under high salt concentration. *PLoS One* **2009**, *4*, e6980, doi:10.1371/journal.pone.0006980.
48. Besir, H.; Zeth, K.; Bracher, A.; Heider, U.; Ishibashi, M.; Tokunaga, M.; Oesterhelt, D. Structure of a halophilic nucleoside diphosphate kinase from *Halobacterium salinarum*. *Febs Lett.* **2005**, *579*, 6595–6600, doi:10.2210/PDB2AZ1/PDB.
49. Tannous, E.; Kanaya, S. Divalent metal ion-induced folding mechanism of RNase H1 from extreme halophilic archaeon *Halobacterium* sp. NRC-1. *PLoS One* **2014**, *9*, e109016, doi:10.1371/journal.pone.0109016.
50. Wende, A.; Johansson, P.; Vollrath, R.; Dyall-Smith, M.; Oesterhelt, D.; Grininger, M. Structural and biochemical characterization of a halophilic archaeal alkaline phosphatase. *J. Mol. Biol.* **2010**, *400*, 52–62, doi:10.1016/J.JMB.2010.04.057.
51. Munawar, N.; Engel, P.C. Overexpression in a non-native halophilic host and biotechnological potential of NAD+-dependent glutamate dehydrogenase from *Halobacterium salinarum* strain NRC-36014. *Extremophiles* **2012**, *16*, 463–476, doi:10.1007/s00792-012-0446-z.
52. Zeth, K.; Offermann, S.; Essen, L.-O.; Oesterhelt, D. Iron-oxo clusters biomineralizing on protein surfaces: structural analysis of *Halobacterium salinarum* DpsA in its low- and high-iron states. *Proc. Natl. Acad. Sci. U. S. A.* **2004**, *101*, 13780–5, doi:10.1073/pnas.0401821101.
53. Holmes, M.L.; Scopes, R.K.; Moritz, R.L.; Simpson, R.J.; Englert, C.; Pfeifer, F.; Dyall-Smith, M.L. Purification and analysis of an extremely halophilic beta-galactosidase from *Haloferax alicantei*. *Biochim. Biophys. Acta* **1997**, *1337*, 276–286.

54. Poidevin, L.; MacNeill, S.A. Biochemical characterisation of LigN, an NAD+-dependent DNA ligase from the halophilic euryarchaeon *Haloferax volcanii* that displays maximal in vitro activity at high salt concentrations. *BMC Mol. Biol.* **2006**, *7*, 44, doi:10.1186/1471-2199-7-44.
55. Pieper, U.; Kapadia, G.; Mevarech, M.; Herzberg, O. Structural features of halophilicity derived from the crystal structure of dihydrofolate reductase from the Dead Sea halophilic archaeon, *Haloferax volcanii*. *Structure* **1998**, *6*, 75–88, doi:10.2210/PDB1VDR/PDB.
56. Britton, K.L.; Baker, P.J.; Fisher, M.; Ruzheinikov, S.; Gilmour, D.J.; Bonete, M.-J.; Ferrer, J.; Pire, C.; Esclapez, J.; Rice, D.W. Analysis of protein solvent interactions in glucose dehydrogenase from the extreme halophile *Haloferax mediterranei*. *Proc. Natl. Acad. Sci. U. S. A.* **2006**, *103*, 4846–4851, doi:10.1073/pnas.0508854103.
57. Kobayashi, T.; Kanai, H.; Aono, R.; Horikoshi, K.; Kudo, T. Cloning, expression, and nucleotide sequence of the alpha-amylase gene from the haloalkaliphilic archaeon *Natronococcus* sp. strain Ah-36. *J. Bacteriol.* **1994**, *176*, 5131–5144.
